# Separation of Permethylated *O*-Glycans, Free Oligosaccharides, and Glycosphingolipid-Glycans Using Porous Graphitized Carbon (PGC) Column

**DOI:** 10.3390/metabo10110433

**Published:** 2020-10-27

**Authors:** Byeong Gwan Cho, Wenjing Peng, Yehia Mechref

**Affiliations:** Department of Chemistry and Biochemistry, Texas Tech University, Lubbock, TX 79409, USA; andrew.cho@ttu.edu (B.G.C.); wenjing.peng@ttu.edu (W.P.)

**Keywords:** glycomics, LC-MS, PGC, glycosphingolipids, free oligosaccharides, *O*-glycans

## Abstract

Glycosylation is one of the most common and complex post-translational modifications of proteins. However, there are other carbohydrates such as free oligosaccharides and glycosphingolipids-glycans that are associated with important biological and clinical roles. To analyze these molecules using liquid chromatography coupled with mass spectrometry (LC-MS), the permethylation approach was utilized. Although permethylation is a commonly utilized glycan derivatization technique, separation of permethylated glycans released from glycosphingolipid (GSL) by LC-MS has never been previously demonstrated. Here, a nanoflow porous graphitized carbon (PGC) column coupled with a high-resolution mass spectrometer was used to achieve isomeric separation of these permethylated glycans. We demonstrate the separation of free reducing end and reduced end *O*-glycans, free oligosaccharides derived from human milk, and GSL glycans derived from the MDA-MB-231BR cancer cell line using PGC-LC-MS.

## 1. Introduction

As a post-translational modification (PTM), glycosylation is one of the most complex and perhaps the most essential modifications of the protein [1]. In fact, its biological functions include immune cell trafficking [2], cell-to-cell adhesion [3] and signaling [4], protein stability [5], and more [6]. Due to the many important physiological roles that glycans play, their importance has been frequently highlighted by researchers from multiple disciplines. Most significantly, glycans’ role in diseases such as cancer [7,8,9,10,11,12], neurodegenerative disorders [13,14], and diabetes [15,16] have been extensively researched. More recently, glycans have been associated with coronaviruses’ spike glycoproteins, including that SARS-CoV-2 virion that causes COVID-19 [17].

Glycosylation is most frequently associated with protein glycosylation, although there are multifarious glycoconjugates, including glycolipids [18,19]. *N*-glycosylation and *O*-glycosylation of proteins are the two major glycoconjugates. *N*-glycosylation involves glycans with a specific core structure being attached to the asparagine, with asparagine-X-serine/threonine amino acid sequence and X being any amino acid (except proline). *O*-glycosylation, on the other hand, occurs on the serine or threonine residue with no specific core structure, yielding a more complex structure [20]. Glycosphingolipids (GSL) are another class of glycoconjugates, wherein a glycan head group is attached to either a ceramide or sphingosine. These can be subclassified into groups such as gangliosides, globosides, and (neo)lactoseries, based on the core structure of their glycan heads. GSL, like glycoproteins, play important biological roles such as those related to immune cell functions [21]. Free oligosaccharides, perhaps the most interesting and abundant carbohydrates in milk, are also involved in the essential biological and clinical functions of the developing immune system in infants [22].

To perform analyses of these complex biomolecules, liquid chromatography coupled with mass spectrometry (LC-MS) is frequently employed to (1) separate convoluted isomeric glycans, (2) detect them with high mass accuracy and sensitivity, and (3) perform fragmentations with the goal of correct structural assignment. The analyses of glycans using LC-MS can differ based on the derivatization techniques utilized for each experiment [23]. For example, reduced native glycans can be analyzed using porous graphitized carbon (PGC) columns [24,25], while permethylated glycans are separated by C18 columns. This approach exploits the hydrophobicity of permethylated glycans [26] and therefore increases ionization efficiency, prompting higher sensitivity [27]. More recently, isomeric separation of permethylated N-glycans using PGC columns has been demonstrated by Zhou et al. to preserve higher sensitivity facilitated by permethylation while delivering isomeric separation [28].

We have reported the efficient isomeric separation of *N*-glycans previously [28,29,30,31]. Here, we demonstrate the separation of smaller glycans such as *O*-glycans, free oligosaccharides, and glycosphingolipid-glycans (GSL-glycans) using permethylation and PGC-LC-MS to resolve isomeric glycans at a high sensitivity. Glycans used in this study have been derived from a variety of biological samples such as bovine fetuin, k-casein, human milk, and the 231BR cancer cell line to demonstrate the applicability of the technique.

## 2. Results

### 2.1. Separation of O-Glycans

**Figure 1:**
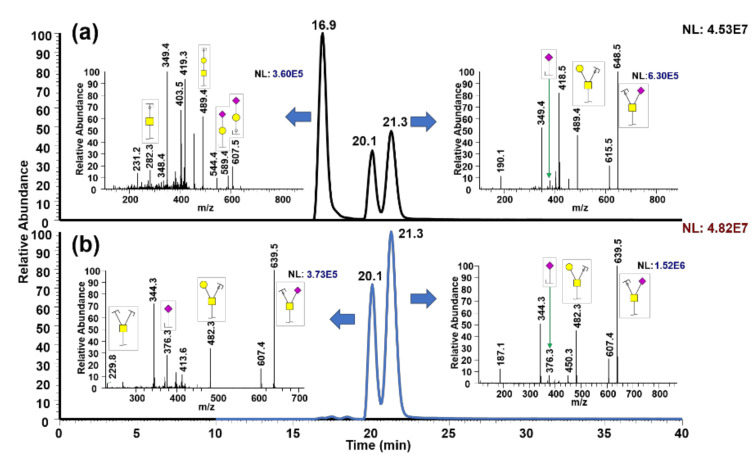
Isomeric separation of permethylated O-glycan isomers derived from k-casein using porous graphitized carbon-liquid chromatography coupled with mass spectrometry (PGC-LC-MS). (**a**) and (**b**) are examples of *O*-glycan isomers: the peak at 16.9 min represents GalNAc-Gal-NeuAc, and the peaks at 20.1 and 21.3 min represent anomers of GalNAc(NeuAc)-Gal. Insets are MS2 spectra of corresponding peaks.

To examine the viability of *O*-glycan separation, *O*-glycans derived from standard glycoproteins were first assessed. Figure 1 shows the separation of permethylated *O*-glycans released from k-casein, with insets depicting MS2 for each isomer. To reduce chromatographic bias, *O*-glycans were multiplexed [32] during the permethylation with ^13^C-labeled methyl iodide, as shown in Figure 1a. Figure 1b depicts permethylated *O*-glycans treated with an exoglycosidase, α2-3 neumeridase, and permethylated with ^12^C methyl iodide. It is worth noting that the *O*-glycans illustrated here are free reducing end form, and therefore α and β anomeric separation was observed. Interestingly, anomeric separation for GalNAc_1_Gal_1_NeuAc_1_ was not observed, although GalNAc1(Gal1)-NeuAc1 displayed anomeric separation.

**Figure 2:**
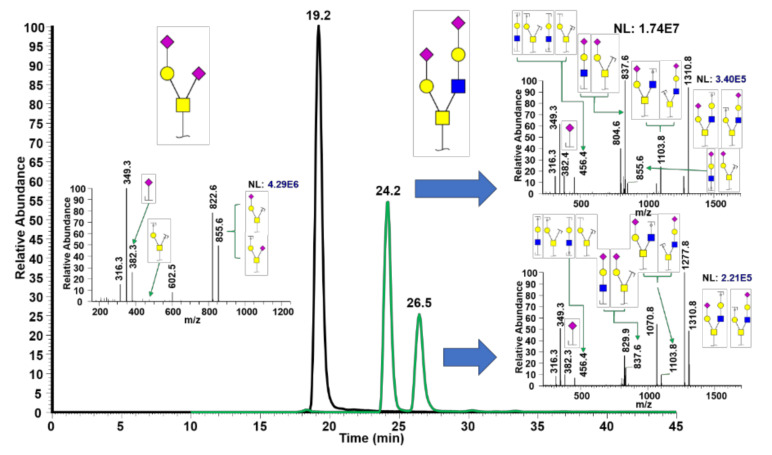
Chromatogram depicting two of the permethylated *O*-glycans derived from bovine fetuin using PGC-LC-MS. Insets are MS2 spectra for the corresponding peaks.

To assess whether anomeric separation would occur in other O-glycans, permethylated O-glycans with free reducing end derived from bovine fetuin were separated using PGC-LC-MS, as shown in Figure 2. GalNAc-Gal-NeuAc showed no anomeric separation; however, GalNAc(Gal-NeuAc)-GlcNAc-Gal-NeuAc exhibited anomeric separation. The MS2 spectra shown for each peak demonstrated that both peaks were the correct structural assignment.

**Figure 3:**
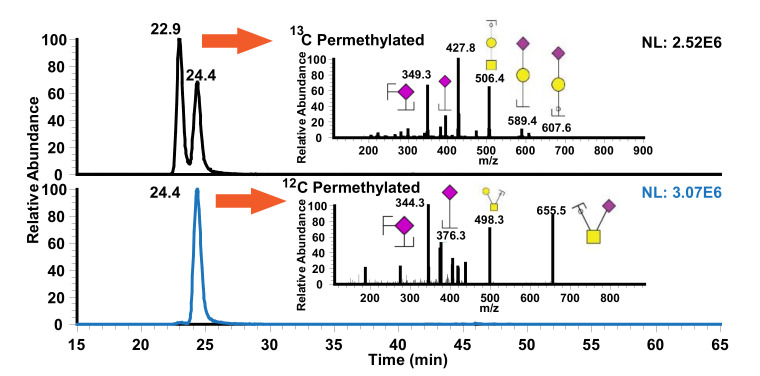
Separation of a reduced trisaccharide *O*-glycan derived from k-casein using PGC-LC-MS. Insets depict MS2 for the corresponding structures.

Expectedly, the determination of isomeric glycans with free reducing ends is more complicated due to unwanted anomeric separation, which results in increased chromatographic complexity. Therefore, reduced reducing end O-glycans were examined with similar experimental approaches to those shown in Figure 1. *O*-glycans derived from k-casein were permethylated with ^12^C methyl iodide, and O-glycans with α2-3 neuraminidase treatment were permethylated with ^13^C, as shown in Figure 3, where the separation of two isomers is clearly demonstrated.

**Figure 4:**
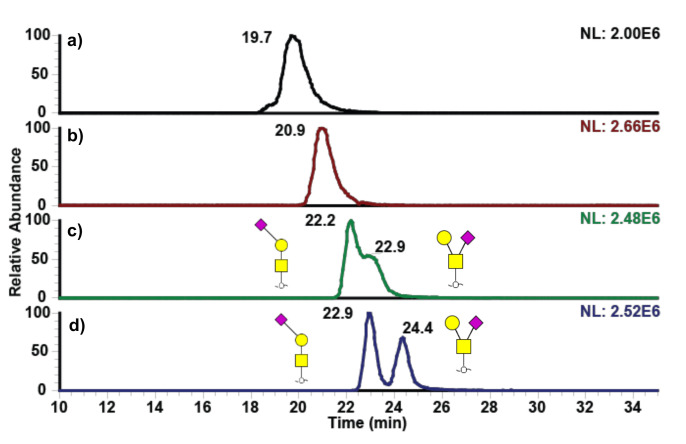
Effect of high temperature in separation of O-glycan. Panel (**a**) separation at 25 °C, (**b**) separation at 45 °C, (**c**) separation at 55 °C, and (**d**) separation at 75 °C.

The effect of column temperature was also assessed, as shown in Figure 4. Permethylated and reduced O-glycans derived from bovine fetuin were analyzed using PGC-LC-MS at four different temperature settings: (a) 25 °C, (b) 45 °C, (c) 55 °C, and (d) 75 °C. Increasing the temperature had a significant effect on peak shape and resolution. As temperature increased, permethylated glycans eluted later, which was in agreement with the previous work by Zhou et al. [28] where permethylated *N*-glycan separation at high temperature was performed.

**Figure 5:**
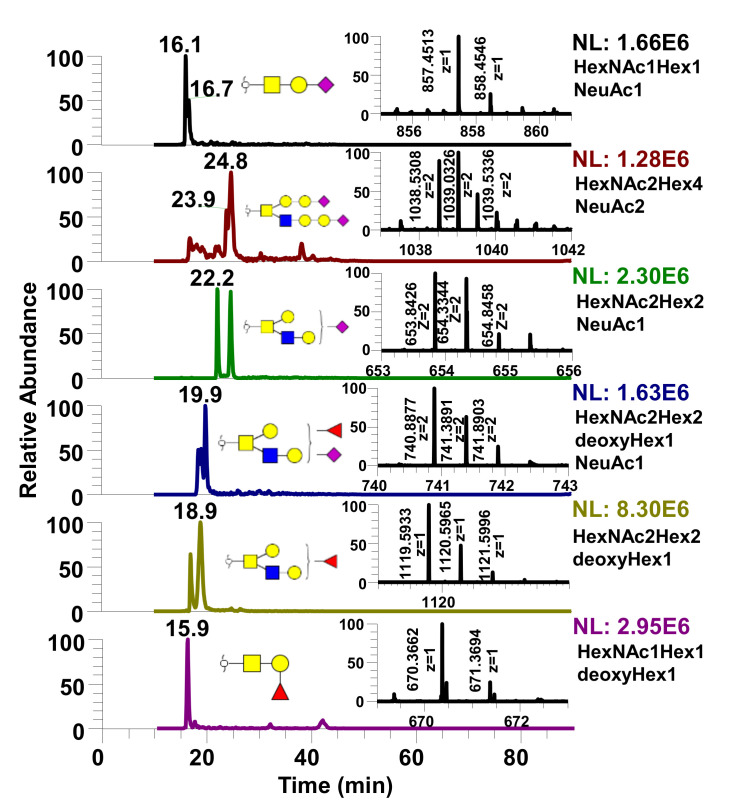
Extracted ion chromatograms (EIC) of various free reducing end *O*-glycans derived from human milk. Insets are mass spectrum of each *O*-glycan structure.

Next, separation of *O*-glycans derived from proteins of human milk was performed using PGC-LC (Figure 5) to show the applicability of the method with biological samples. It is noteworthy that the chromatograms shown in Figure 5 represent free reducing end *O*-glycans. This approach was chosen due to increased sensitivity as a result of the enzymatic/chemical release of *O*-glycans demonstrated by Goetz et al. [33].

### 2.2. Separation of Free Oligosaccharides and Glycosphingolipid Glycans

**Figure 6:**
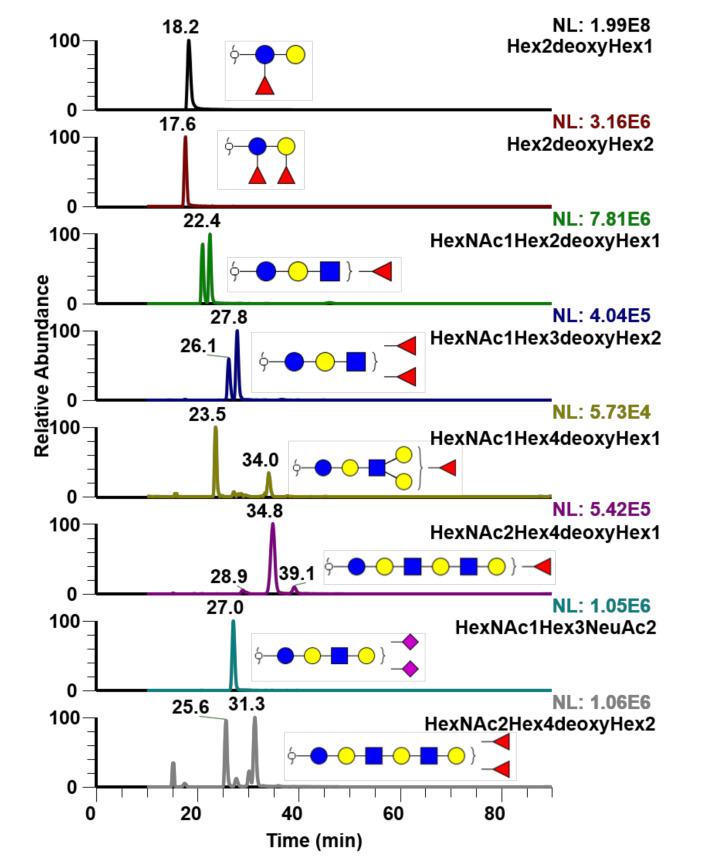
EIC (Extracted ion chromatogram) of free oligosaccharides extracted from human milk. Isomeric separation of some free oligosaccharides was achieved using PGC-LC-MS. MS2 spectra of the displayed structures were depicted in Figures S1–S7.

As previously mentioned, free oligosaccharides and GSL-glycans are other important glycoconjugates in biological and physiological systems. An analysis of free oligosaccharides from human milk is shown in Figure 6. Unlike *O*-glycans, these are free sugars and are not attached to any other bioconjugates, so no release step was needed. Therefore, the free oligosaccharides were reduced and then permethylated to avoid anomeric separation. Notably, separation of focusylated oligosaccharides was achieved using this technique.

**Figure 7:**
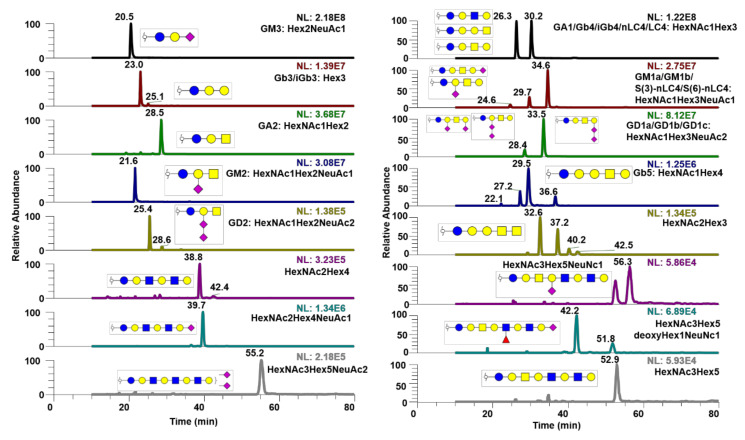
EIC of various glycosphingolipid (GSL) glycans derived from the 231BR cell line. Isomers of GSL-glycans are depicted with captions of their possible identity. MS2 spectra of the displayed structures were depicted in Figures S8–S16.

Furthermore, GSL-glycans were analyzed using PGC-LC-MS, as shown in Figure 7. In this case, GSL-glycans derived from breast cancer cell line 231BR were analyzed using PGC-LC-MS. Endoglycoceramidase I was utilized to release the glycans from GSL, which were then purified, reduced, and permethylated prior to analysis. The glycan head group from GSL was then analyzed.

## 3. Discussion

In this work, we demonstrated isomeric separation of permethylated *O*-glycans, free oligosaccharides, and GSL-glycans using a PGC-LC technique that has been previously shown to separate permethylated *N*-glycans [28,29,30,31]. Several separation techniques for *O*-glycans were used, including the utilization of ion mobility [34] and PGC-LC [34,35] for analysis of native *O*-glycans. Interestingly, in this study, anomeric as well as isomeric O-glycan separation was observed, as shown in Figure 1; Figure 2. Furthermore, GalNAc(NeuNc)-Gal and GalNAc(GlcNAc-Gal-NeuAc)Gal-NeuAc showed anomers being separated, while GalNAc-Gal-NeuAc and GalNAc(NeuAc)-Gal-NeuAc did not show anomers. To confirm this assertion, MS2 spectra, exoglycosidase, α2-3 neuraminidase, and multiplexing permethylation were employed (Figure 1 and Figure 2). However, we note that glycan anomers are biologically irrelevant. The anomerization of alpha and beta anomers on the reducing ends of the glycan is a reversable process, once glycans are liberated from glycoproteins or other glycoconjugates. Not only are anomeric glycans irrelevant, they also interfere with the identification of glycan isomers by complicating chromatograms, making it impossible to pinpoint whether a given peak is an isomer or an anomer. Therefore, we examined permethylated reduced reducing end O-glycans derived from k-casein, as shown in Figure 3. Using the same technique applied in Figure 1 and Figure 2, effective separation of sialic acid-linked *O*-glycan isomers was demonstrated. The peak at 22.9 min indicates GalNAc-Gal-NeuAc while the peak at 24.4 min indicates GalNAc(NeuAc)-Gal, as validated using both MS2 and α2-3 neuraminidase. It is important to mention the intensity differences between non-reduced *O*-glycans (Figure 1) and reduced *O*-glycans (Figure 3), where non-reduced *O*-glycans show more than 10 times the intensity of reduced *O*-glycans. This observation was in agreement with the previous study from Goetz et al. [33], where the intensity of *O*-glycans was 10 times higher compared to reduced and permethylated O-glycans, after using protease and pronase to digest the glycoprotein followed by permethylation to liberate and permethylate the O-glycans simultaneously.

While the analysis of permethylated *N*- and *O*-glycans using PGC-LC-MS was initially performed by Cipollo et al. [36], separation of sialylated O- and N-glycans were not demonstrated. However, our work demonstrates the analysis of permethylated sialylated *O*-glycans at high temperatures. Using high temperature for the separation of permethylated N-glycans was previously demonstrated by Zhou et al. [28]. Utilizing the similar approach, the effect of PGC column temperature for separation of permethlyated *O*-glycan was investigated, as shown in Figure 4. Increased temperature drastically improved the peak shape and resolution of positional isomers, GalNAc-Gal-NeuAc and GalNAc(Gal)-NeuAc. This result revealed that permethylated O-glycans have a similar effect as permethylated N-glycans when separated on PGC column at high temperature.

Due to the increase in intensity, *O*-glycans were derived from human milk using an enzymatic/chemical method, as shown in Figure 5. As mentioned above, when both anomers and isomers were present in the sample, it was difficult to distinguish the two since some free reducing end O-glycans show anomeric separation while some do not. Therefore, researchers face a tradeoff between preserving sensitivity or maintaining simple chromatography for better identification of glycan isomers.

Next, free oligosaccharides from human milk were analyzed using PGC-LC-MS (Figure 6). It is worth noting that free oligosaccharides from human milk were the result of simply collecting the flow through a 10k molecular weight cut-off (MWCO) filter. Proteins collected with the filter were utilized to derive the O-glycans shown in Figure 5. Analysis of free oligosaccharides from human milk has been performed most recently by Porfirio et al. [37], and in previous research by Mank et al. [38], Xu et al. [39], and Dong et al. [40]. Mank et al. and Xu et al. selected a PGC column to analyze native reduced free oligosaccharides, while Porfirio et al. utilized a C18 reverse phase column to analyze permethylated free oligosaccharides without showing isomers of the free oligosaccharides. Dong et al. used both C18 and PGC columns to analyze the free oligosaccharides from different milk sources, however only one compositional glycan showing two isomers was exemplified. As shown in Figure 6, various isomers of each free oligosaccharide are depicted. It is important to note, however, that the oligosaccharides shown here are reduced reducing end oligosaccharides. Therefore, there are no anomers complicating the chromatograms.

Lastly, GSL-glycans derived from the 231BR cancer cell line were separated using PGC-LC, as shown in Figure 7. This is the first time that permethylated GSL-glycans have been analyzed using an LC-MS system, others have preferred 2-aminobenzamide (2-AB) labeling [41] or native [42] glycan analysis, although permethylation of intact glycosphingolipids [43] has been previously demonstrated. This work reveals that permethylated GSL-glycan isomers can be separated using PGC-LC while preserving the sensitivity that permethylation delivers to mass spectrometers. However, identification of these isomers can be tricky due to the lack of structural differences between some of the GSL-glycans. For example, there are four possible GSL-glycan isomers for HexNAc1Hex3. The only difference between neolacto4 and lacto4 is the beta linkage on the outer galactose. Nevertheless, PGC-LC allows separation of permethylated GSL-glycan isomers. For future work, it might be intriguing to investigate GSL-glycan profiles of different cancer cell lines or biological fluids from patients of various diseases, as well as to assign glucose units for these glycans, as shown by Gautam et al. [26]. The biggest pitfall of this technique may be the fact that the column used in this work is no longer produced by ThermoFisher. However, some researchers have already demonstrated that in-house packed PGC columns work just as well, as shown by Xiao et al. [44].

## 4. Materials and Methods

Bovine fetuin, k-casein, trifluoracetic acid, borane-ammonia complex, methyl iodide, methyl iodide (^13^C), and sodium hydroxide beads were purchased from Sigma-Aldrich. High performance liquid chromatography (HPLC) water was obtained from Avantor. Methanol, acetonitrile, dimethyl sulfoxide (DMSO), and ammonium hydroxide were acquired from Fisher Scientific. Formic acid was from Tokyo Chemical Industry. 10k cut-off membrane filter units were purchased from MilliporeSigma. The C18 solid phase extraction cartridge and PGC column were from ThermoFisher (Thermo Fisher Scientific, Waltham, MA, USA). Activated charcoal spin columns and C8 spin columns were purchased from Harvard Apparatus. Endoglycocermidase I and α2-3 Neuraminidase S were bought from New England Biolabs. Pronase was purchased from Roche. Cell line MDA-MB-231BR was a generous gift from Dr. Paul Lockman (West Virginia University, School of Pharmacy, Morgantown, WV, USA). The Dulbecco’s Modified Eagle Medium (DMEM) and fetal bovine serum (FBS) for cell lines were purchased from ATCC. Trypsin-ethylenediaminetetraacetic acid (EDTA) 1X (0.25% Trypsin/2.21 mM EDTA) was purchased from Corning.

### 4.1. O-Glycan Release

Free reducing end O-glycans from bovine fetuin and k-casein were prepared by a chemical/enzymatic approach. 10 µg of each glycoprotein was subjected to pronase digestion (Protein:Enzyme = 10:1) by incubating at 55 °C for 48 h. Then, the samples were dried and subjected to solid phase permethylation. Dried pronase digested samples were resuspended in 30 µL of DMSO, 1.2 µL of water, and 20 µL iodomethane (either ^12^C or ^13^C, depending on the experiment). Resuspended samples were then added to the sodium hydroxide bead-packed micro spin column and incubated for 25 min at room temperature. An additional 20 µL of methyl iodide was added, then incubated for another 15 min at room temperature. After the incubation, spin columns were centrifuged at 1.8k g for 2 min at room temperature. Next, 50 µL of acetonitrile was added to each spin column then centrifuged again at the same speed for 2 min. Collected permethylated glycans were dried in the SpeedVac prior to analysis.

Reduced reducing end O-glycans were prepared according to the protocol by Huang et al. [45]. 10 µg of glycoproteins were resuspended in 5 mg/mL borane-ammonia complex in ammonium hydroxide and incubated for 18 h at 45 °C. After incubation, the samples were dried in the SpeedVac and methanol was added to discard the borane. Finally, the samples were permethylated as described above.

### 4.2. α2-3. Neuraminidase S Digestion

For the samples with α2-3 neuraminidase S digestion, protocols from New England Biolabs were followed exactly. 5 µg of glycoproteins were resuspended in 45 µL of water, 5 µL of Glycobuffer1, and 5 µL of α2-3 neuraminidase S and incubated for 1 h at 37 °C. Depending on the experiment, free reducing end *O*-glycan or reduced reducing end *O*-glycan procedures were followed as described above.

### 4.3. Preparation of O-Glycans and Free Oligosaccharides from Human Milk

The human milk sample was filtered through a 10k MWCO filter (MiliporeSigma, Burlington, MA, USA). The flow through was collected for free oligosaccharide analysis, and the proteins collected in the filter were used for O-glycan analysis according to the procedures described above. Free oligosaccharides were reduced by the addition of 10 mg/mL borane-ammonia complex with incubation for 1 h at 60 °C. Methanol washes were performed until no white residues remained, and samples were permethylated as described above.

### 4.4. Cell Line Cultivation

Cell line MDA-MB-231BR (231BR) was stored in liquid nitrogen. One tube (1 mL) of stored 231BR was taken out, thawed in a 37 °C water bath, and transferred to a 75 cm^2^ flask. Then, 11 mL of DMEM (with 20% FBS and 2% penicillin-streptomycin solution) was added to the flask. The cells were then incubated at 37 °C with 5% CO_2_ for four days. During incubation, cells were fed every two days. After reaching an 80% cell confluence, cells were washed with 10 mL phosphate buffer saline (PBS). Next, cells were detached by adding 2.2 mL of trypsin-EDTA solution and incubating for 5 min at 37 °C. After incubation, 5 mL of fresh medium was added and mixed to neutralize the trypsin, followed by inoculating 1 mL to a 175 cm^2^ flask (three 175 cm^2^ flasks were prepared as three triplicates). Then, 24 mL of complete DMEM was added to the flask, and cells were incubated at 37 °C, 5% CO_2_ for 5 days until an 80% cell confluence was reached. Cells were then washed with 20 mL PBS and detached with 5 mL trypsin-EDTA solution, as described above. After detachment, 10 mL of fresh medium was added and mixed. The cell culture was transferred to a 15 mL tube and centrifuged at 500× *g* for 5 min at room temperature. The cell pellet was washed two times with PBS to remove the remaining medium, and then stored at −20 °C for further analysis.

### 4.5. GSL Extraction from 231BR Cell Line

Cell lysate was subjected to lipid extraction using methanol and chloroform adopted from Albrecht et al. [41]. 100 µL of chloroform and 200 µL of methanol was added to 75 µL of cell lysate, vortexed and sonicated for 5 min, then centrifuged at 14 k g for 2 min. Supernatant was collected and a mixture of chloroform and methanol (1:1) was added to the pallet, vortexed, sonicated, and centrifuged. The supernatant was pooled and the same steps were repeated with a mixture of chloroform and methanol (2:1). Pooled supernatant was dried in the SpeedVac, then a C8 spin column purification of glycosphingolipids was performed according to Benktander et al. [46]. Dried lipids were resuspended in a methanol and water mixture (3:2). C8 spin columns were washed with methanol three times, conditioned with a methanol and water mixture (3:2) twice, then the resuspended lipid sample was added to the spin column and centrifuged. Eluate was added back to the column and centrifuged, then the columns were washed with a methanol and water mixture (3:2) three times. Glycosphingolipids were eluted by applying methanol twice and drying in the SpeedVac.

### 4.6. GSL-Glycan Preparation

Dried GSL were resuspended in 14 µL of water and 4 µL of endoglycoceraminidase I (EGCase I) Buffer. 2 µL of endoglycoceramidase I was added to the sample and incubated at 37 °C for 18 h. After the incubation, the samples were dried in the SpeedVac and resuspended in 5% acetic acid (aq) for C18 purification. The C18 cartridges (1 mL) were washed with methanol three times and conditioned with 5% acetic acid (aq) three times. Samples were applied to the cartridges and flow through was collected. 5% acetic acid (aq) was applied three more times, pooled into the flow-through vial, and dried. Dried GSL-glycans were resuspended in 0.1% trifluoroacetic acid (TFA) in 85% acetonitrile and 15% water to perform solid phase extraction using activated charcoal spin columns. Spin columns were washed with 0.1% TFA in 85% acetonitrile and 15% water three times, then conditioned with 0.1% TFA in 5% acetonitrile and 95% water three times. Samples were applied to the columns and centrifuged, then eluants were reapplied to the column. This step was repeated twice. Next, the columns were washed with 0.1% TFA in 5% acetonitrile and 95% water twice before elution of glycans with 0.1% TFA, 50% acetonitrile, and 50% water twice. Purified GSL-glycans were reduced as described in Section 4.3 and permethylated as described in Section 4.1.

### 4.7. PGC-LC-MS

Online purification of permethylated glycans was performed using a PepMap C18 Trapping Column (0.075 mm ×2 cm, 3 µm) which was equipped on an UltiMate 3000 RSLCNano system (Thermo Fisher Scientific, Waltham, MA, USA) connected to an LTQ Orbitrap Velos (Thermo Fisher Scientific, Waltham, MA, USA). The analytical column was the Thermo Scientific PGC (Thermo Fisher Scientific, Waltham, MA, USA) Hypercarb (0.075 mm ×10 cm, 5 µm). Flow rate was set at 600 nL/min with temperature set at 75 °C. Mobile phase A was 0.1% formic acid in water, and mobile phase B was 0.1% formic acid in acetonitrile. The gradient was kept at 20% B for the first 10 min while online purification occurred. Then, the valve was switched to load purified permethylated glycans onto the analytical column at 10 min. B was set to 60% at 25 min then increased to 95% at 46 min and kept for 38 min. B was decreased to 20% for re-equilibration for 16 min. The LTQ Orbitrap Velos was set at 1.6 kV, positive mode, with full MS performing at 100,000 resolution and scan range from 400 to 2000 *m/z*. Data-dependent acquisition was performed with collision-induced dissociation (CID) settings set to 5000 minimum signal required, 30 normalized collision energy, and 0.25 activation Q, and the top 6 peaks were subjected to fragmentation.

### 4.8. Data Processing

Raw files were processed manually using Xcalibur 4.2 (Thermo Fisher Scientific, Waltham, MA, USA). Glycans were annotated with the assistance of Glycoworkbench 2. The table of contents was made with BioRender.com.

## 5. Conclusions

In this work, we demonstrated for the first time the use of PGC-LC-MS at high temperature to separate permethylated *O*-glycans, free oligosaccharides, and released GSL-glycans from cell lines. Our work showed the capability of PGC column for separating permethylated *O*-glycans, free oligosaccharides, as well as GSL-glycans. This work also investigated anomeric and isomeric separation of *O*-glycans. This method could be used to analyze isomeric *O*-glycans on antibody-based therapeutics but also could be utilized to examine the role of *O*-glycans in cancer and other diseases. The use of PGC-LC-MS for the analysis of permethylated free oligosaccharide and GSL-glycan isomers facilitated extensive isomeric separation as well as enhanced sensitivity allowed by enhanced ionization via permethylation. This technique can be adopted to further investigate GSL-glycan composition and their isomers derived from other cancer cell lines to examine potential glycolipid-based cancer biomarkers with the emphasis on the glycan head group.

## Supplementary Materials

The supplementary materials are available online at https://www.mdpi.com/2218-1989/10/11/433/s1.
