# Supplementary material for: Separation of Permethylated O-Glycans, Free Oligosaccharides, and Glycosphingolipid-Glycans Using Porous Graphitized Carbon (PGC) Column
Source: Metabolites. 2020 Oct 27;10(11):433. doi: 10.3390/metabo10110433 (PMC7692250; doi:10.3390/metabo10110433)
Supplement: Supplementary file 1 [file metabolites-10-00433-s001.pdf]

Supplementary Information

Separation of Permethylated *O*-Glycans, Free Oligosaccharides, and Glycosphingolipid-Glycans Using Porous Graphitized Carbon (PGC) Column

Byeong Gwan Cho, Wenjing Peng and Yehia Mechref *

Department of Chemistry and Biochemistry, Texas Tech University, Lubbock, TX, 79409, USA; andrew.cho@ttu.edu (B.G.C.); wenjing.peng@ttu.edu (W.P.)

* Correspondence: yehia.mechref@ttu.edu

Received: 20 September 2020; Accepted: 21 October 2020; Published: date

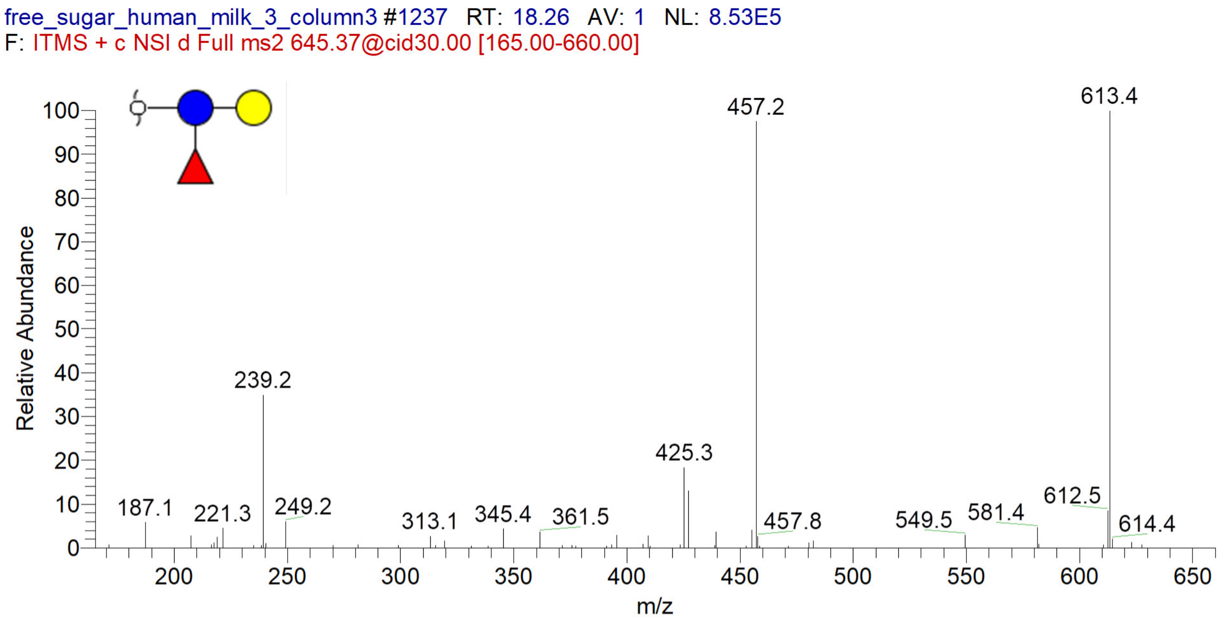

Figure S1. MS2 spectra of Hex2deoxyHex1 derived from human milk.

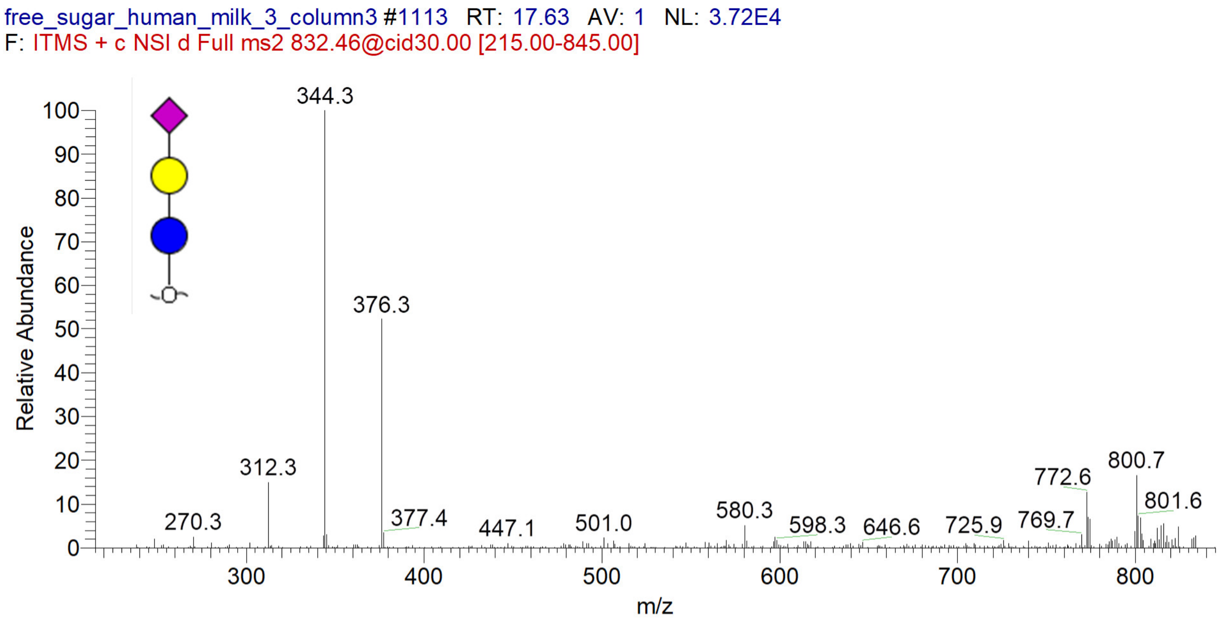

Figure S2. MS2 spectra of Hex2NeuAc1 derived from human milk.

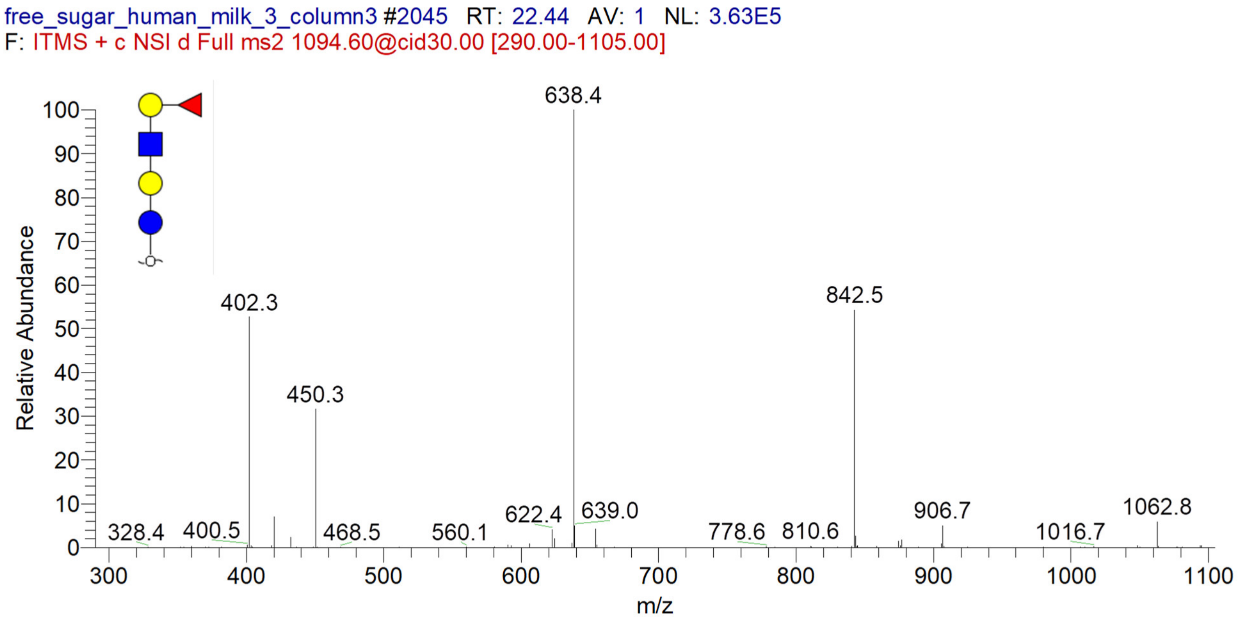

Figure S3. MS2 spectra of HexNAc1Hex3deoxyHex1 derived from human milk.

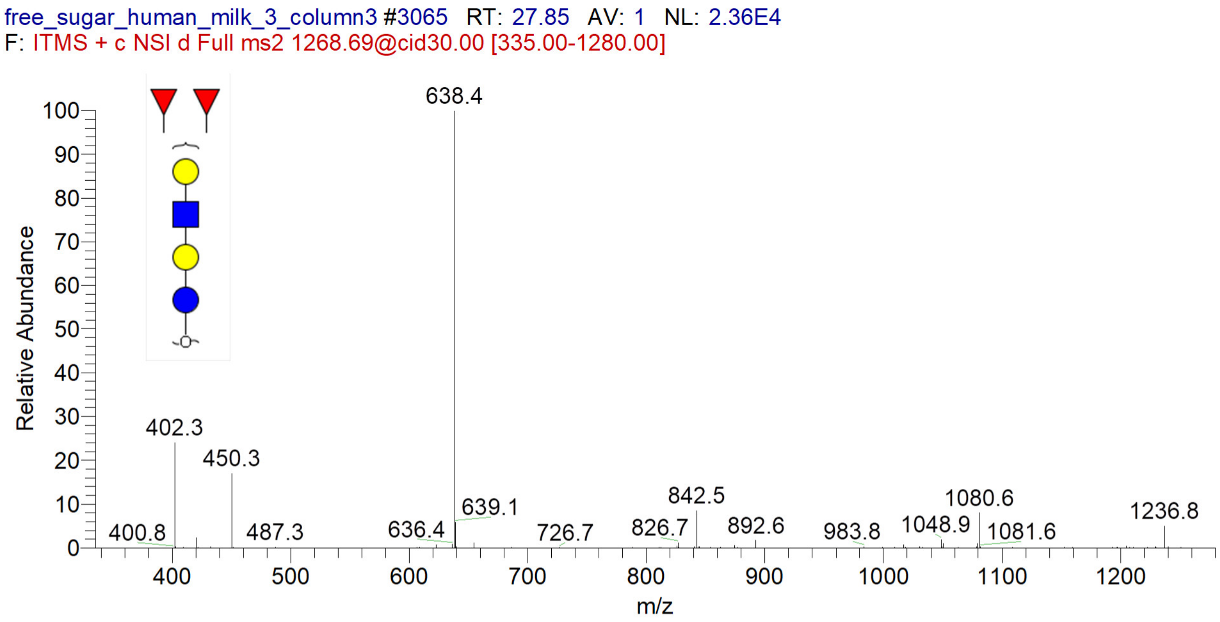

Figure S4. MS2 spectra of HexNAc1Hex3deoxyHex2 derived from human milk.

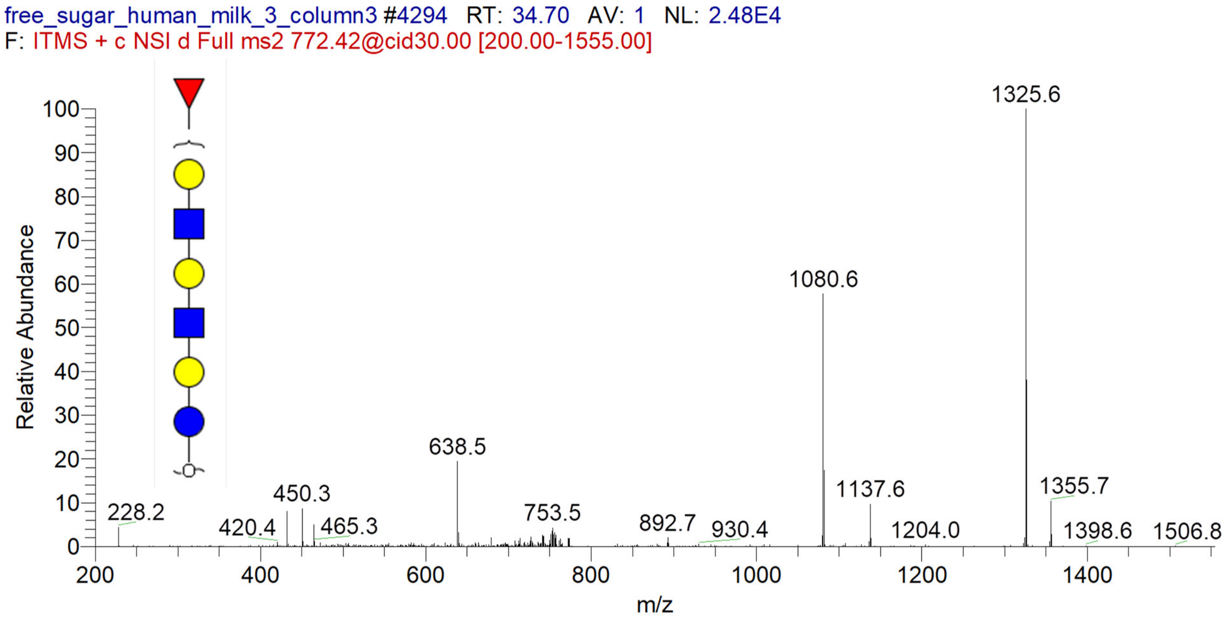

Figure S5. MS2 spectra of HexNAc2Hex4deoxyHex1 derived from human milk.

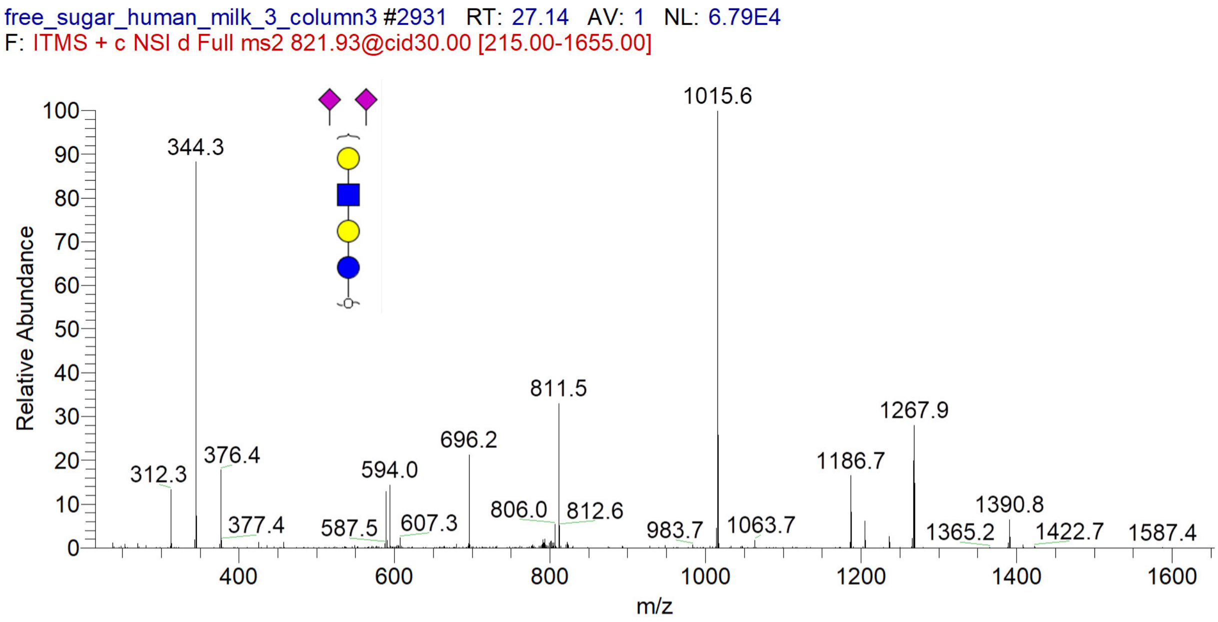

Figure S6. MS2 spectra of HexNAc1Hex3NeuAc2 derived from human milk.

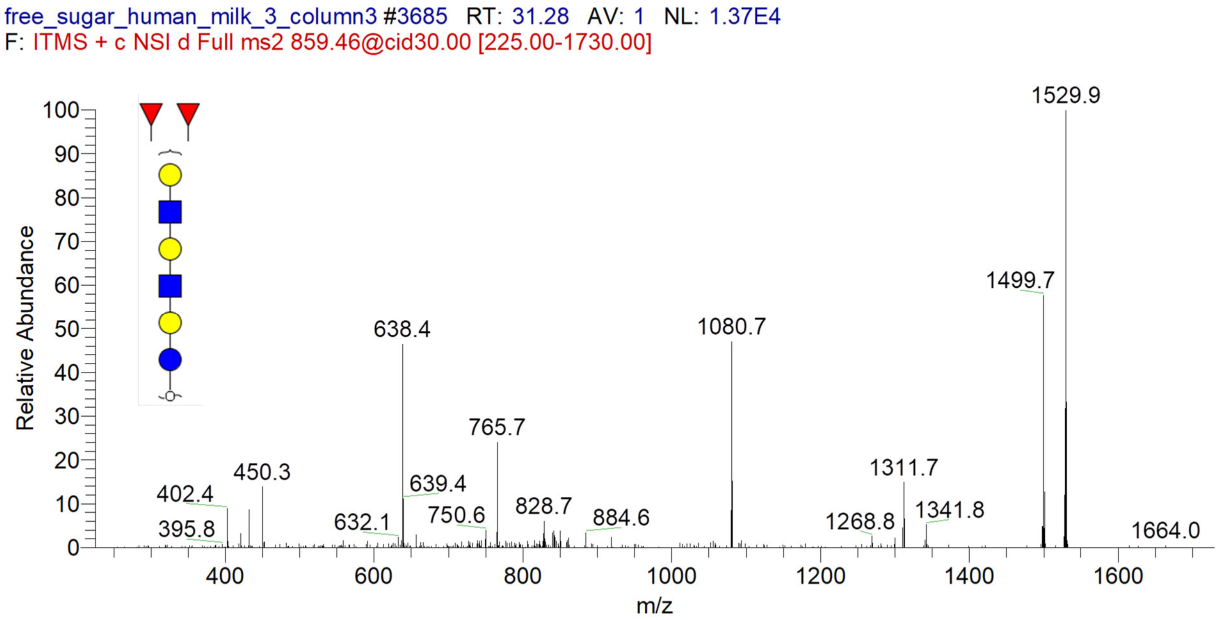

Figure S7. MS2 spectra of HexNAc2Hex4deoxyHex2 derived from human milk.

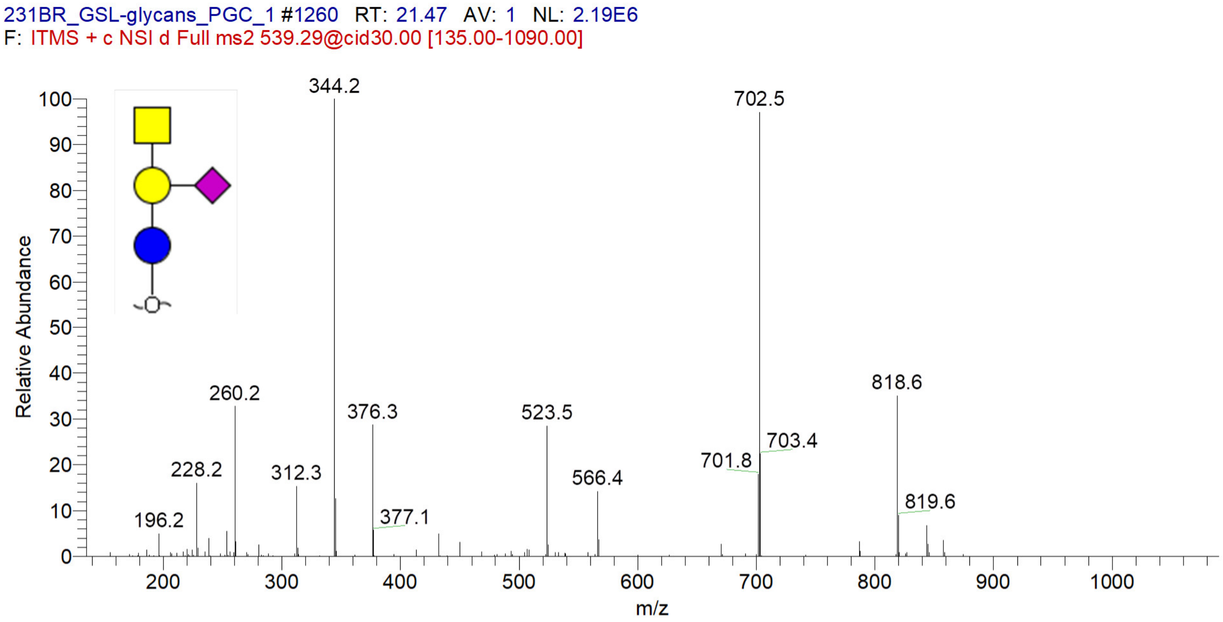

Figure S8. MS2 spectra of HexNAc1Hex2NeuAc1 released from 231BR cell line glycolipid.

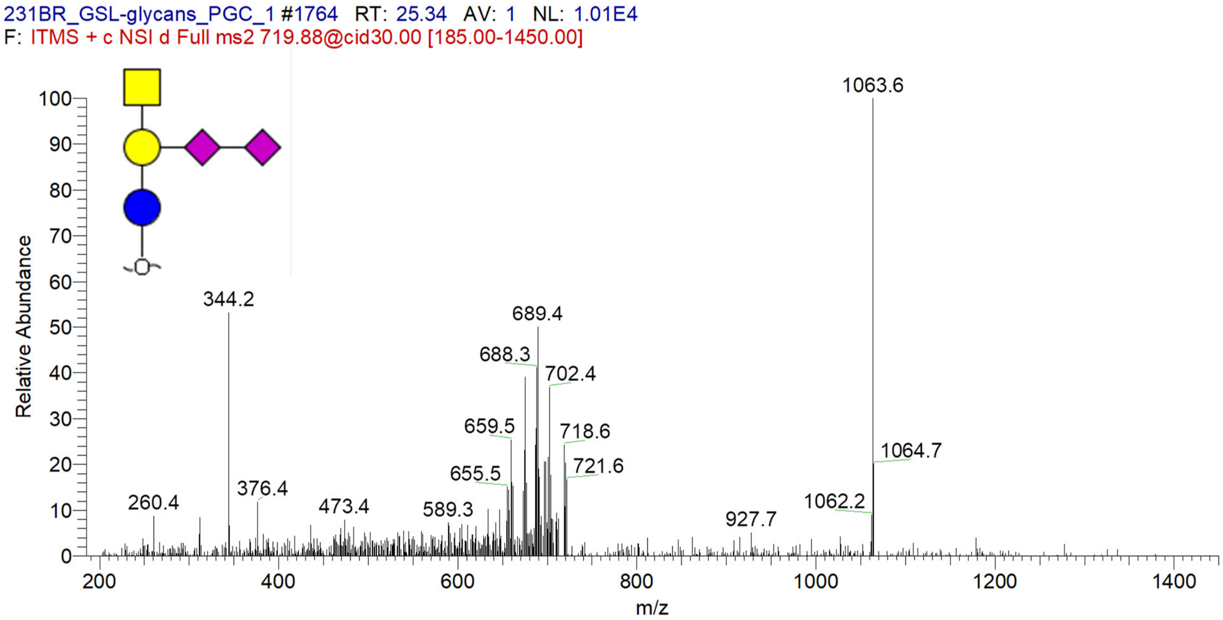

Figure S9. MS2 spectra of HexNAc1Hex2NeuAc2 released from 231BR cell line glycolipid.

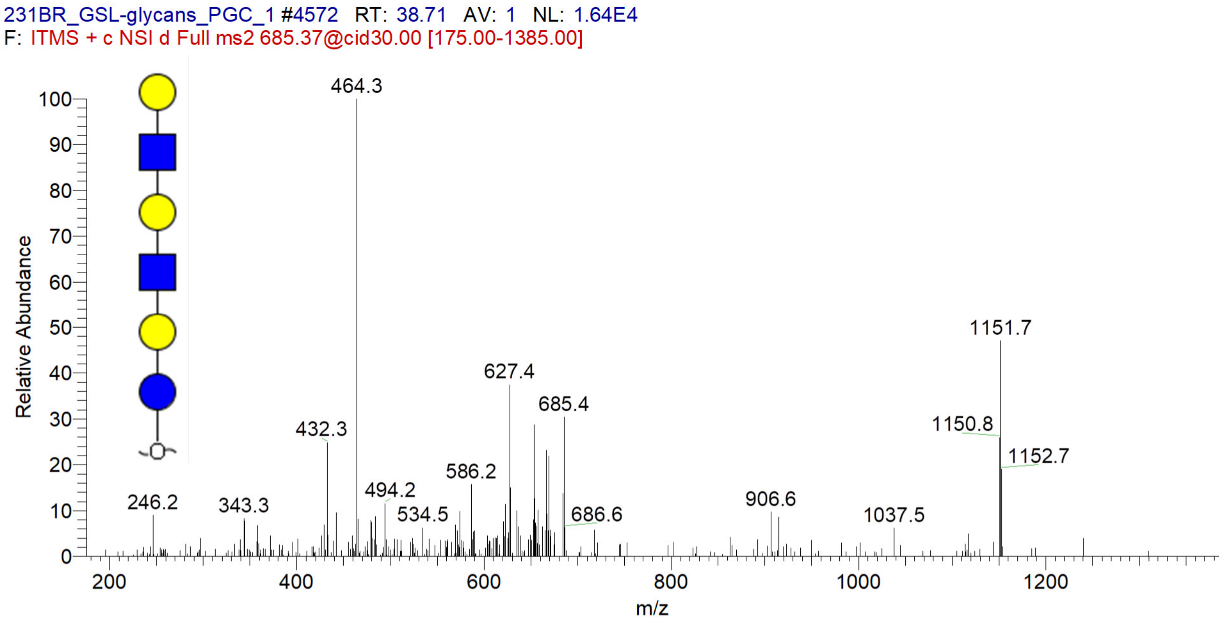

Figure S10. MS2 spectra of HexNAc2Hex4 released from 231BR cell line glycolipid.

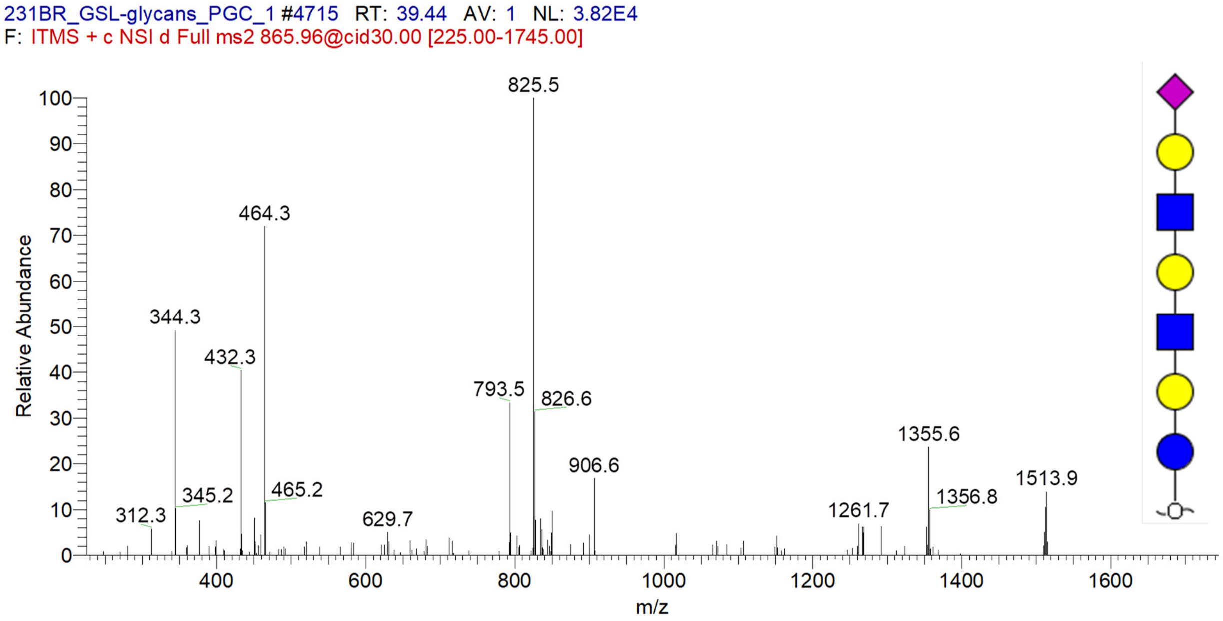

Figure S11. MS2 spectra of HexNAc2Hex4NeuAc1 released from 231BR cell line glycolipid.

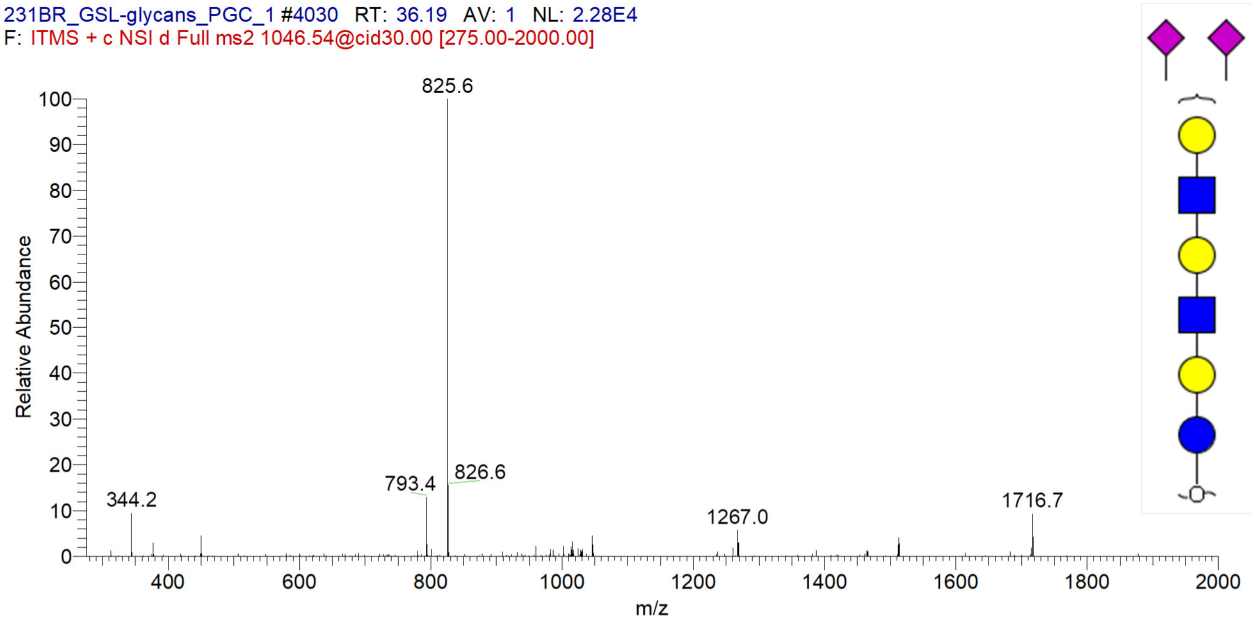

Figure S12. MS2 spectra of HexNAc2Hex4NeuAc2 released from 231BR cell line glycolipid.

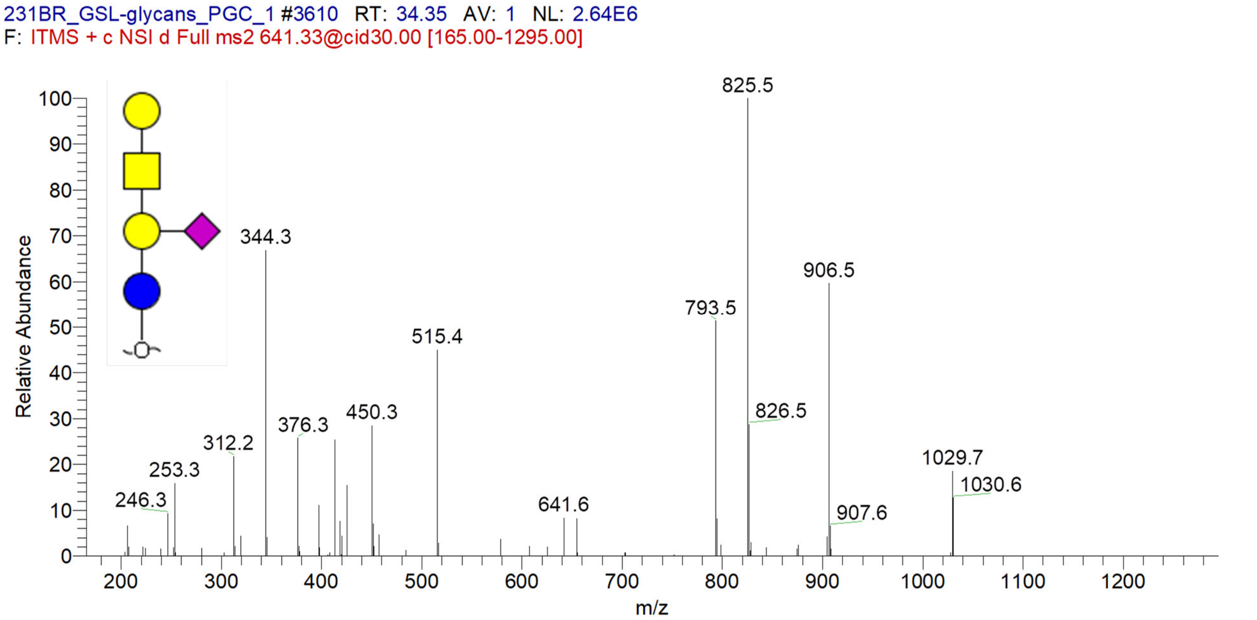

Figure S13. MS2 spectra of HexNAc1Hex3NeuAc1 released from 231BR cell line glycolipid.

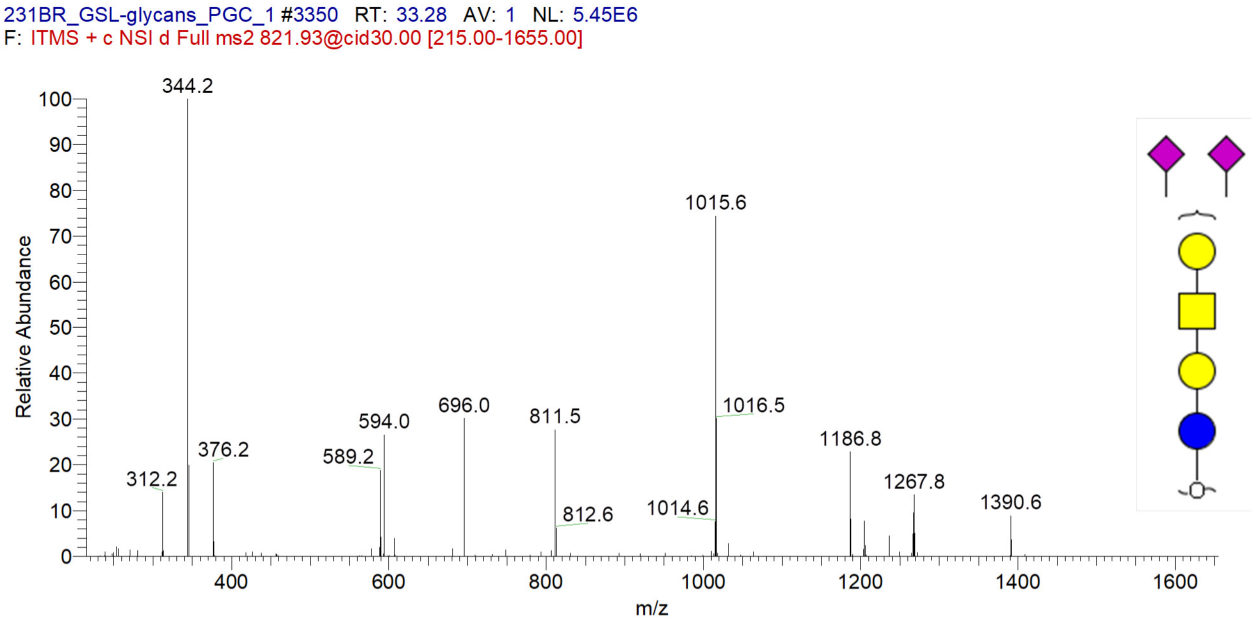

Figure S14. MS2 spectra of HexNAc1Hex3NeuAc2 released from 231BR cell line glycolipid.

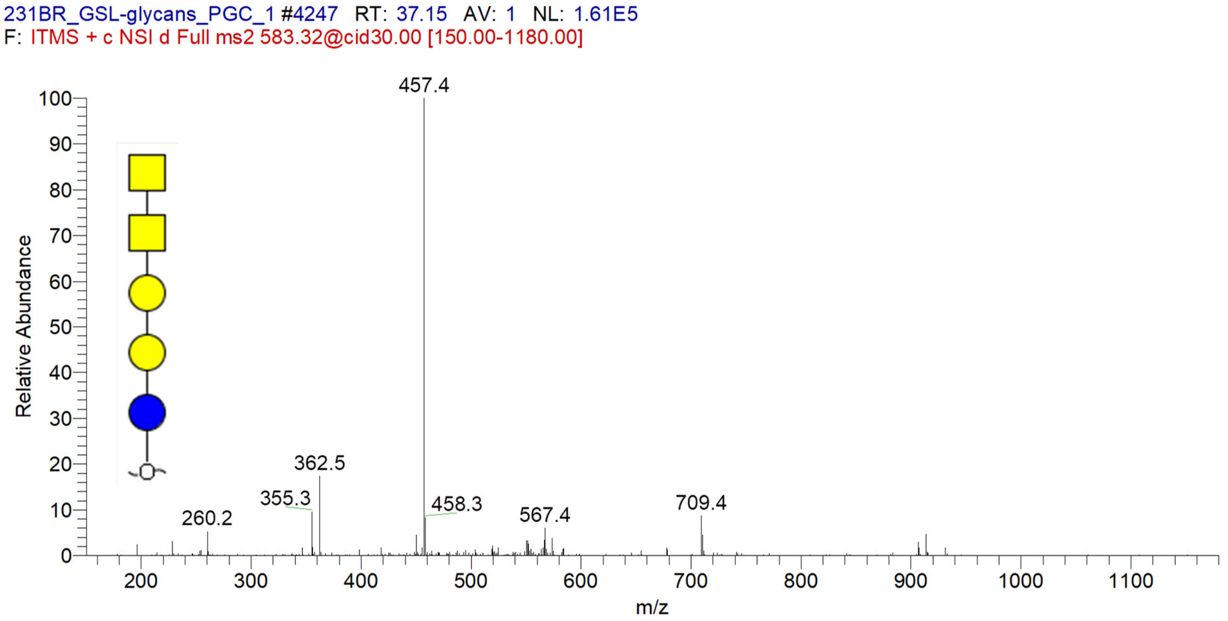

Figure S15. MS2 spectra of HexNAc2Hex3 released from 231BR cell line glycolipid.

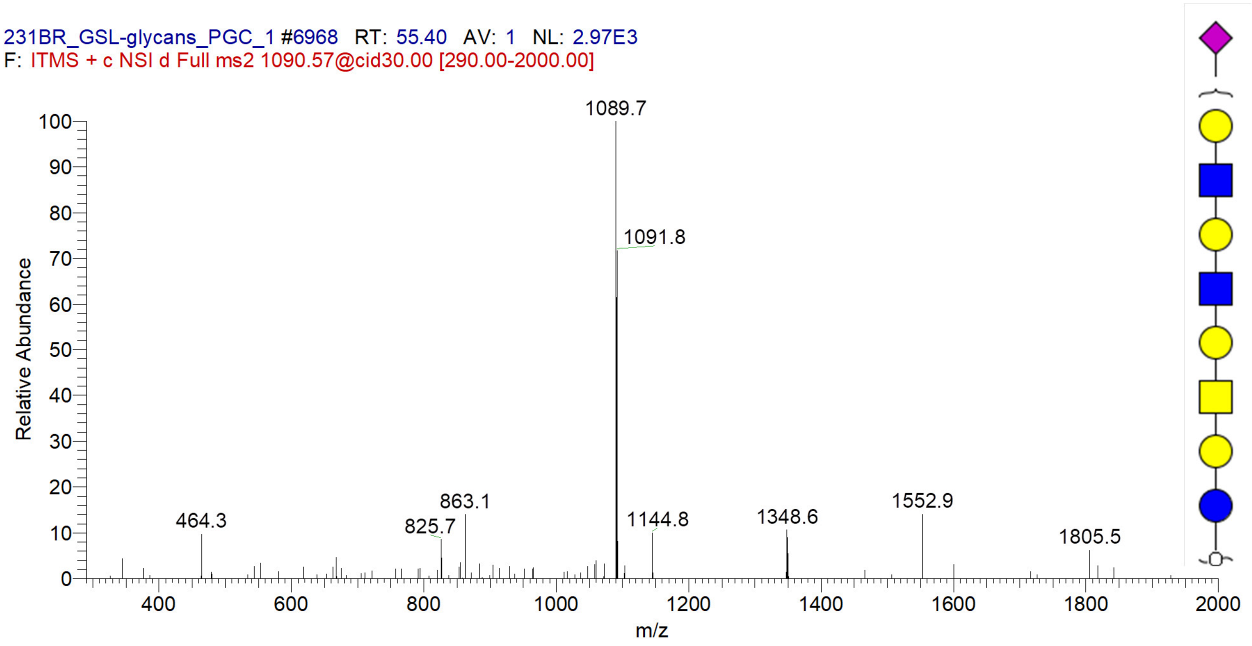

Figure S16. MS2 spectra of HexNAc3Hex5NeuAc1 released from 231BR cell line glycolipid.
